# Circulating immune cells and vitiligo: a bidirectional two-sample Mendelian randomization study

**DOI:** 10.3389/fimmu.2024.1391186

**Published:** 2024-06-03

**Authors:** Yu Xin, Tao Yuan, Jun Wang

**Affiliations:** Department of Dermatology, Yijishan Hospital affiliated with Wannan Medical College, Wuhu, China

**Keywords:** vitiligo, original article, Mendelian randomization, circulating immune cells, immune

## Abstract

**Background:**

The pathogenesis of vitiligo remains elusive. Emerging evidence suggests that vitiligo is an immune-mediated disorder, in which a plethora of immune cells play pivotal roles. However, the association between circulating immune cells and vitiligo continues to be enigmatic.

**Materials and methods:**

We extracted single nucleotide polymorphisms (SNPs) associated with immune circulating cells at a genome-wide significance level from the BLOOD CELL CONSORTIUM’s genome-wide association study (GWAS) dataset. Summary data for 385,801 cases of vitiligo were obtained from a large-scale Finnish genome-wide association study (ncases=292, ncontrols=385,509). The inverse variance weighted (IVW) method was employed as the primary analytical approach for Mendelian randomization (MR) analysis. Additionally, heterogeneity was assessed using Cochran’s Q value, and horizontal pleiotropy was evaluated using MR-Egger Mendelian Randomization Pleiotropy RESidual Sum and Outlier and leave-one-out analyses.

**Results:**

The risk of vitiligo was found to increase with the elevation of 4 circulating immune cells, as evidenced by the odds ratios (ORs) and 95% confidence intervals (CIs): basophils (OR=1.81; 95% CI: 1.01–3.24, p=0.0450), monocytes (OR=1.67; 95% CI: 1.23–2.26, p=0.0009), eosinophils (OR=1.78; 95% CI: 1.22–2.59, p=0.0028), and neutrophils (OR=1.65; 95% CI: 1.08–2.54, p=0.0208). After removing outliers, the sensitivity analysis of the above indicators did not show heterogeneity and pleiotropy.

**Conclusion:**

Our findings illuminate the association between circulating immune cells and vitiligo, offering insights that could guide clinical practices in the treatment of vitiligo.

## 1 Introduction

Vitiligo is characterized as a chronic skin condition where there is a loss of pigment, leading to white patches appearing on the skin. The primary manifestation of vitiligo is the appearance of irregular white patches on the skin, which can occur on any part of the body Vitiligo affects a significant number of individuals globally, with estimates ranging from 0.5% to 2.0% of the world population, 39 million to 156 million individuals may be affected by this condition, making it the most common pigmentary disorder worldwide (1). While vitiligo can start at any age, over 60% of those affected begin showing symptoms before they turn 30, indicating a tendency towards earlier onset. It impacts both sexes equally, though a slightly higher incidence is observed in females (2, 3). Beyond the physical symptoms, vitiligo carries a significant social stigma that can severely impact an individual’s quality of life. This stigma can lead to decreased self-esteem, social isolation, and psychological stress, which are compounded by the visible nature of the disorder (4).

In the studies of vitiligo, immune cells in both the skin and the peripheral blood play a critical role in the pathogenesis of the disorder (5). The research into vitiligo often involves case studies or experimental models which, while valuable, come with inherent limitations. Many of these studies can be affected by confounding factors, where other variables affect both the conditions being studied and the outcomes. Moreover, issues like reverse causation bias, where it is unclear whether the condition causes the observed effect or vice versa, can complicate interpretations of results. These factors can lead to contradictions in research findings, making it difficult to draw definitive conclusions and to develop universally effective treatments. For example, regulatory T cells (Tregs) are crucial for maintaining immune homeostasis by suppressing overly aggressive responses that could damage the body. They typically inhibit the activity of other T cell types, such as CD4+ and CD8+ T cells (6). Yet, in a single-cell transcriptomics study, Tregs from the peripheral blood of vitiligo patients exhibited proinflammatory characteristics instead of immunosuppressive properties, such as increased sensitivity to interferon (5). According to a study, In individuals with vitiligo, there is a noticeable reduction in the number of invariant NKT (iNKT) cells (7). However, a study showed these cells can play a dual role in immune responses, being either protective or pathogenic depending (8). The inconsistent results from previous studies indicate the need for a novel approach to elucidate the connection between the two.

Mendelian Randomization (MR) is a powerful technique in genetic epidemiology that leverages genetic variants, such as single nucleotide polymorphisms (SNPs), to act as proxies or instrumental variables in determining potential causal relationships between various exposures and outcomes (9). The fundamental principle behind Mendelian Randomization is the random assortment of alleles at conception, which segregates the genetic variants independently of the postnatal environment and disease state. This natural randomization acts akin to the random assignment in a controlled trial, where the genotype serves as an unconfounded proxy for the exposure. By examining genetic determinants that are fixed at conception and therefore not influenced by disease processes or environmental factors that could introduce bias. MR provides a more robust assessment of causality, helping to avoid the pitfalls of confounding factors and reverse causation that often plague observational studies. In this study, MR is utilized to dissect the genetic underpinnings linking immune cell levels to the development of vitiligo.

## 2 Materials and methods

### 2.1 Study design

**Figure 1:**
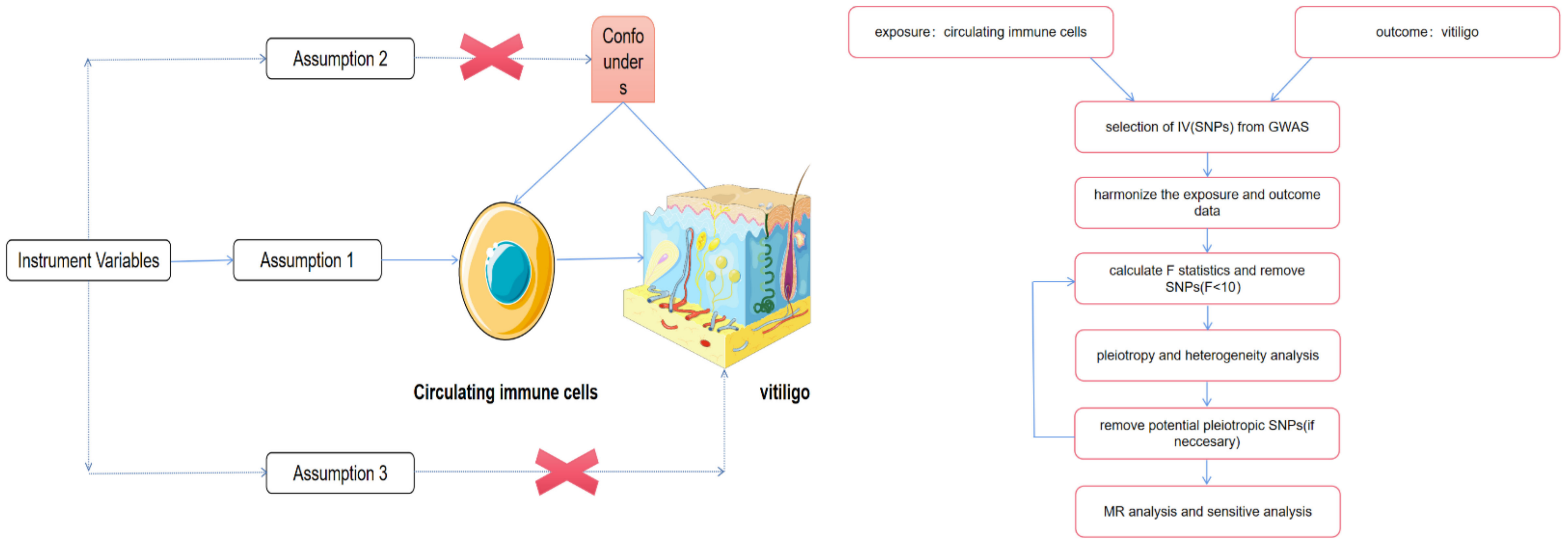
The schematic illustration of the causal relationship between circulating immune cells and vitiligo through MR analyses. Parts of the figure were drawn by using pictures from Servier Medical Art. Servier Medical Art by Servier is licensed under a Creative Commons Attribution 4.0 Unported License (https://creativecommons.org/licenses/by/4.0/). No changes made.

To accurately infer the potential causal relationship between circulating immune cells and vitiligo using the MR approach, three criteria must be satisfied: (1) The genetic variants should be significantly associated with the exposure; (2) When used as instrumental variables for the exposure, the genetic variants should not be associated with any confounders; (3) The genetic variants should affect the outcome solely through their impact on the exposure (10, 11). **Figure 1** depicts the flowchart of the MR study process. Since all data were sourced from open-access public databases, ethical review was not required.

### 2.2 Data sources

We obtained publicly available summary statistics from genome-wide association studies (GWAS) for MR analysis. Data on 6 circulating immune cells were derived from a GWAS conducted by the BLOOD CELL CONSORTIUM (sample size=563,946), accessible through the IEU Open GWAS database, which contained 26 discovery cohorts of European ancestry additional to the UK Biobank, totaling 563,946 subjects. The log10-transformed absolute count, in units of (×10^9^ cells/L), were used in the BCX2 GWAS after multiplying relative counts with the total WBC count from the original cohorts. Summary statistics for vitiligo were sourced from the tenth release of FinnGen, with a sample size of 385,801 (ncases=292, ncontrols=385,509) (12). The Finnish GWAS database is a specialized bioinformatics resource designed to collect and integrate genome-wide association study data from the Finnish population. This database capitalizes on the distinctive genetic architecture of the Finnish population, characterized by its significant homogeneity and unique regional genetic bottlenecks. These features make it an exemplary cohort for the study of genetic diseases and traits, providing a valuable resource for understanding the complexities of genetics in a well-defined population group. In these studies, all case and control cohorts were of European ancestry, with no significant overlap between the GWAS populations. Specific details can be found in **Table 1**.

**Table 1:** Characteristics of the Circulating Immune Cells and Vitiligo GWAS.

Phenotype	Ancestry	Participants	CONSORTIUM	ieu ID/phenocode
Basophil cell count	European	563,946	Blood cell consortium	ieu-b-29
White cell count	European	563,946	Blood cell consortium	ieu-b-30
Monocyte cell count	European	563,946	Blood cell consortium	ieu-b-31
Lymphocyte cell count Eosinophil cell count Neutrophil cell count Vitiligo	European European European European	563,946 563,946 563,946 385,801	Blood cell consortium Blood cell consortium Blood cell consortium NA	ieu-b-32 ieu-b-33 ieu-b-34 L12_VITILIGO

### 2.3 IV selection

To ensure the robustness of MR analyses, genetic instrumental variables (IVs) for exposures were selected based on stringent genome-wide significance thresholds (*p* < 5 × 10^-8). Given the potential for linkage disequilibrium (LD) to introduce bias into MR analyses, it is imperative to address LD to ensure the independence of single nucleotide polymorphisms (SNPs). The parameters were set as follows to mitigate the effects of LD: r^2 = 0.001 and kb = 10,000. This approach underscores the importance of rigorous selection criteria and LD adjustment in the construction of genetic instruments to facilitate unbiased causal inference in genetic epidemiology (13). Concurrently, palindromic sequences were excluded from our analysis to enhance the integrity of the genetic data. To detect potential biases arising from weak instrumental variables, we calculated the proportion of variance explained (R^2) and the F-statistic for all single nucleotide polymorphisms (SNPs). Instrumental variables with an average F-statistic greater than 10 were incorporated into our study (14).

### 2.4 Statistical analysis

To ascertain the causal relationship between circulating immune cells and vitiligo, our study employed MR analysis utilizing four distinct approaches: Inverse Variance Weighted (IVW), MR Egger, Weighted Median, and Weighted Mode. The IVW method served as the primary analytical tool, with the other methods applied as supplementary analyses to validate and reinforce the robustness of our findings (15). In our study, the Inverse Variance Weighted (IVW) method employs a fixed-effect meta-analysis model to integrate the causal effect estimates from each SNP on the relationship between circulating immune cells and vitiligo. This approach assumes homogeneity in the causal effect across all SNPs, leveraging the precision of each SNP’s estimate to provide a weighted average effect size. The fixed-effect model is particularly suited for scenarios where the genetic instruments are believed to exert uniform effects on the exposure and subsequently on the outcome, minimizing the influence of between-study heterogeneity (16). Mr. Egger’s role is to identify any instances of pleiotropy and address any potential bias (17). The weighted median is supplemented by MR Egger, ensuring robust results are maintained even in the presence of 50% invalid instrumental variables (18). The weighted mode method may have lower efficacy in identifying causality, but it helps lower the likelihood of Type I errors (19). In our analysis, the results obtained from the IVW method are considered the primary outcomes, with the findings from the other three methods—MR Egger, Weighted Median, and Weighted Mode—serving as supplementary evidence. When p < 0.05, we consider the exposure to be significantly associated with the outcome.

Subsequently, we implemented sensitivity analyses to address and mitigate biases stemming from heterogeneity and pleiotropy, ensuring a more robust and reliable interpretation of our findings. Cochran’s Q test was employed to assess the heterogeneity of causal estimates between exposure and outcome, with a p-value <0.05 considered indicative of significant heterogeneity (16). Furthermore, the MR Egger intercept test was applied to determine the presence of horizontal pleiotropy, and MR-PRESSO analysis was utilized to identify and correct for outliers (20). Lastly, a “leave-one-out” analysis was conducted to ascertain the stability of the results and to identify potential heterogeneity among SNPs. Furthermore, we applied the Bonferroni correction to adjust for multiple testing. The Bonferroni correction of the evidential threshold was based on the number of exposures, setting a *p*-value threshold of < 0.05 divided by the number of exposures (6), resulting in a corrected threshold. Thus, an association with a two-sided *p*-value < 0.0083 (0.05/6 exposures) was deemed statistically significant, while a two-sided *p*-value < 0.05 was considered suggestive of an association (21).

All statistical analyses in this study were performed using the R software (version 4.3.2). MR analysis was conducted using the Two sample MR package (version 0.5.8) in R. *p* < 0.05 is considered significant, 0.0083<*p* < 0.05 is deemed suggestive.

## 3 Results

In the MR analysis, the IVW results indicated that the risk of vitiligo increases with the elevation of basophils (Odds Ratio [OR]=1.81; 95% Confidence Interval [CI]: 1.01–3.24, p=0.0450), monocytes (OR=1.67; 95% CI: 1.23–2.26, p=0.0009), and neutrophils (OR=1.65; 95% CI: 1.08–2.54, p=0.0208). Although conclusions drawn from the other three methods may differ from the IVW results.

**Figure 2:**
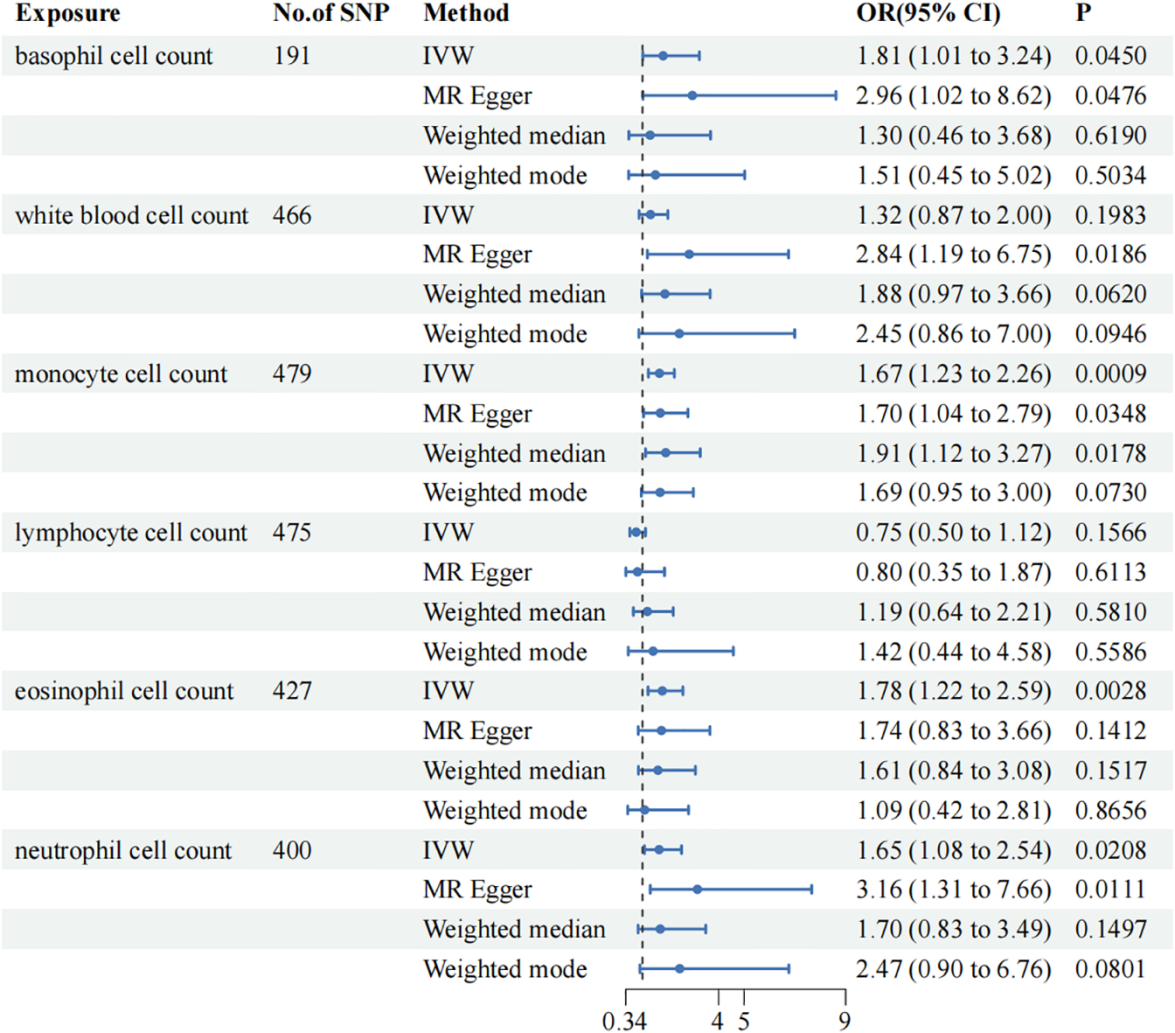
Mendelian Randomization (MR) association between genetically predicted circulating immune cells and vitiligo. Odds ratios (ORs) are scaled to the risk of vitiligo for genetically predicted increases in circulating immune cells. CI, confidence interval.

**Figure 3:**
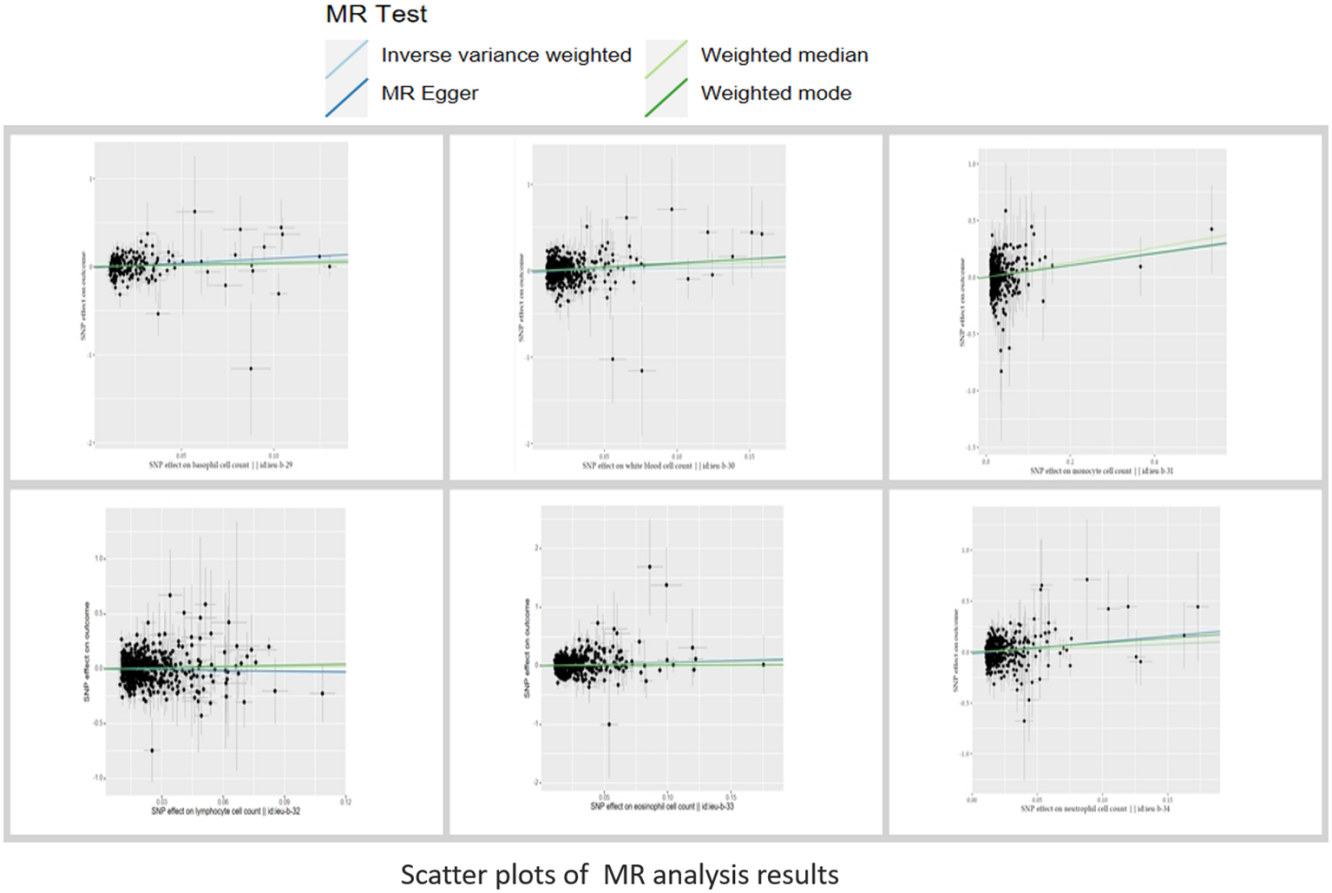
Scatter plots of the causal effects of circulating immune cells on vitiligo.

To ensure the reliability of our study results, we conducted several sensitivity analyses. Cochran’s Q test indicated no heterogeneity in the primary outcomes. The MR-Egger intercept did not detect any potential horizontal pleiotropy (p > 0.05). Using MR-PRESSO analysis, SNPs with heterogeneity were identified. After removing the relevant SNPs, eosinophils showed a causal relationship with vitiligo (Odds Ratio [OR]=1.78; 95% Confidence Interval [CI]: 1.22–2.59, p=0.0028). Sensitivity analyses were conducted again, and only white blood cells exhibited heterogeneity and pleiotropy. Even after applying the Bonferroni correction, the causal relationships between monocytes and eosinophils with vitiligo were still significant, whereas the associations of basophils and neutrophils with vitiligo were suggestive.MR Analysis Results are presented in **Figure 2**. Sensitivity Analysis Results are presented in **Table 2**. Scatter plots are shown in **Figure 3**.

**Table 2:** Summary of sensitivy results.

Phenotype	Q	Q_pval	MR-PRESSO Global Test	MR-Egger intercept	pval	Filtered SNP(s)
Basophil cell count	198.1565	0.3276	0.3243	0.0147	0.2834	NA
White cell count	546.7775	0.0052	0.0050	0.0204	0.0477	NA
Monocyte cell count	475.0074	0.5301	0.5373	0.0008	0.9136	NA
Lymphocyte cell count	524.3746	0.0545	0.0570	0.0019	0.8526	“rs10774624”
eosinophil cell count	457.7427	0.1391	0.1380	0.0006	0.9529	“rs1265564” “rs7382061”
neutrophil cell count	409.6341	0.3458	0.3410	0.0169	0.1023	NA

In the reverse MR analysis, due to the scarcity of available SNPs under the original selection criteria (p < 5 × 10^-8), we relaxed the exposure criteria to (p < 5 × 10^-5). The results of the reverse MR analysis indicated no significant association between the two. See **Table 3** for MR analysis and **Table 4** for sensitivity test. More details are contented in **Supplementary Materials**.

**Table 3:** Results of reverse MR analysis.

Outcome	Exposure	NO.SNPs	Method	*p*	*β*	95%CI	Outliers
Basophil cell count	vitiligo	32	IVW MR-Egger Weighted Median Weighted Mode	0.3973 0.2729 0.3261 0.5009	-0.0009 -0.0030 -0.0016 -0.0018	-0.003,0.001 -0.008,0.002 -0.005,0.002 -0.007,0.003	NA
White cell count	vitiligo	31	IVW MR-Egger Weighted Median Weighted Mode	0.9538 0.0067 0.5744 0.2846	9.59E-05 -0.1000 -0.0010 0.0034	-0.003,0.003 -0.017–0.003 -0.002,0.004 -0.003,0.009	“rs139359727”“rs148460321”“rs34104137”
Monocyte cell count	vitiligo	31	IVW MR-Egger Weighted Median Weighted Mode	0.7624 0.1799 0.523 0.5116	-0.0003 -0.0034 -0.0010 -0.0017	-0.002,0.002 -0.008,0.001 -0.004,0.002 -0.007,0.003	NA
Lymphocyte cell count	vitiligo	31	IVW MR-Egger Weighted Median Weighted Mode	0.1989 0.9339 0.1050 0.6618	0.0017 0.0003 0.0026 0.0010	-0.001,0.004 -0.006,0.007 -0.001,0.006 -0.004,0.006	NA
eosinophil cell count	vitiligo	31	IVW MR-Egger Weighted Median Weighted Mode	0.3098 0.5112 0.6509 0.9656	0.0012 -0.0018 0.0007 9.04E-05	-0.001,0.003 -0.007,0.004 -0.002,0.004 -0.004,0.004	NA
neutrophil cell count	vitiligo	31	IVW MR-Egger Weighted Median Weighted Mode	0.9000 0.0038 0.4670 0.5505	-0.0002 -0.0112 0.0012 0.0017	-0.004,0.003 -0.018,-0.004 -0.002,0.005 -0.004,0.007	“rs139359727”“rs34104137”“rs9859558”

**Table 4:** Summary of sensitivy results of reverse MR.

Phenotype	Q	Q_pval	MR-PRESSO Global Test	MR-Egger intercept	pval	Outlier
Basophil cell count	30.8620	0.4732	0.4563	0.0012	0.4054	NA
White cell count	78.9953	2.75E-06	<0.0003	0.0058	0.0030	“rs139359727” “rs148460321” “rs34104137”
Monocyte cell count	29.4332	0.4949	0.5063	0.0018	0.1797	NA
Lymphocyte cell count	50.2913	0.0116	0.01233	0.0008	0.6225	NA
eosinophil cell count	34.6629	0.2551	0.2690	0.0017	0.2357	NA
neutrophil cell count	82.0442	1.00E-06	<0.0003	0.0064	0.0020	“rs139359727” “rs34104137” “rs9859558”

## 4 Discussion

To the best of our knowledge, this study is the inaugural effort to employ MR to elucidate the potential causal relationship between circulating immune cells and vitiligo. Our findings reveal a significant association between the two, offering additional theoretical insights that could enhance the clinical diagnosis and treatment of vitiligo.

Vitiligo is an autoimmune disorder where the immune system mistakenly targets and destroys melanocytes, which are responsible for pigment production in the skin. This targeted attack leads to a progressive loss of skin color. Research has shown that CD8+ T cells, when isolated from the lesions of vitiligo-affected skin and cultured *in vitro*, are capable of killing melanocytes even from normally pigmented areas of the same individual. The ability of CD8+ T cells to specifically target and kill melanocytes in vitiligo patients suggests that these immune cells are both necessary and sufficient for the progression of the disease (22). Autoreactive cytotoxic CD8 T cells interact with melanocytes and contribute to the advancement of the disease by producing Interferon γ (IFN-γ) in the local area. Subsequently, keratinocytes surrounding the area secrete chemokines induced by IFN-γ, which, through a positive feedback loop, further recruit T cells to the skin.

Basophils are granulocytes with a diameter of 5 to 8 μm, characterized by segmented and condensed nuclei. They typically constitute <1% of the peripheral white blood cells in the blood (23). Basophils in humans contribute to immune responses by releasing histamine, T helper (Th)2 cytokines, and various other mediators. A study found an increase in the expression of genes associated with Th2 responses (such as IL-13, CCL5, and CCL18) in the skin of patients (24). In a bioinformatics analysis, the number of activated CD4+ and CD8+ T cells and Th2 cells was significantly increased in vitiligo lesions compared to control skin samples (25). This provides an explanation for the potential role of basophils in vitiligo.

Eosinophils are traditionally considered terminally differentiated cytotoxic effector cells. Mature eosinophils are released into the peripheral blood and enter tissues in response to synergistic signaling between IL-5 and the eotaxin family of chemokines (26). Eosinophils participate in Type II immune responses, which are associated with T helper 2 cells and various interleukins (27). Eosinophils contribute to tumor rejection by facilitating the normalization of tumor vasculature and augmenting the infiltration of CD8 T cells. This effect is attributed to the secretion of IFN-γ, TNF, CCL5, CXCL9, and CXCL10 by eosinophils, underscoring their pivotal role in orchestrating anti-tumor immune responses (28). This may also offer a potential explanation for the involvement of eosinophils in vitiligo. In summary, eosinophils play a significant role in vitiligo; however, the precise mechanisms warrant further investigation.

Blood monocytes are essential components of the immune system, originating from common myeloid progenitors (CMPs) in the bone marrow. CMPs are versatile stem cells that also differentiate into several other crucial blood cells, including erythrocytes (red blood cells), platelets, conventional dendritic cells (cDCs), and granulocytes. Once monocytes are produced, they are released into the peripheral bloodstream (29). Regulatory T (Treg) cells, recognized for their expression of the FOXP3 transcription factor, are pivotal in maintaining immune homeostasis. GWAS in vitiligo have highlighted regulatory gene features linked to these cells, underscoring their role in suppressing the autoimmune responses that characterize vitiligo (30). A set of reports indicates that in patients with vitiligo, Treg cells exhibit a compromised ability to control the activity of CD8 T cells, which are instrumental in the autoimmune attack on melanocytes. This reduction in suppressive capacity observed *in vitro* suggests that an impairment in Treg cell functionality could be a critical factor contributing to the autoimmune pathology of vitiligo, allowing for unchecked T cell-mediated destruction of pigment cells (31). In vitiligo, melanocyte-reactive CD8 T cells demonstrate an escape from anergy—a state in which autoreactive T cells are normally non-responsive to prevent self-damage. This escape indicates a failure in the immune system’s checks and balances, permitting these T cells to actively attack melanocytes (32). A previous study has shown that the proportion of CD80+ monocytes is significantly elevated in the peripheral blood of individuals with vitiligo compared to healthy controls. CD80 is a costimulatory molecule that enhances T cell activation and response. This increase suggests that monocytes may contribute to the heightened immune response seen in vitiligo, although their function has not yet been explored (33). In a single-cell transcriptomics study, monocytes from vitiligo patients have higher expression levels of genes related to inflammatory cytokines, chemokines, and transcription factors. Such findings highlight the inflammatory role of monocytes in vitiligo, suggesting that they may be active participants in the disease’s progression by fostering an environment conducive to autoimmune reactions against melanocytes (5).

Neutrophils are the predominant type of white blood cells in human peripheral circulation and are essential for our immune defense system. These cells are primarily known for their ability to rapidly respond to pathogenic infections and sterile inflammation. Despite their short lifespan, typically lasting only a few days, neutrophils are incredibly effective at containing and eliminating pathogens through various mechanisms, including phagocytosis, degranulation, and the release of antimicrobial peptides (34). A previous study indicates that neutrophils in patients with vitiligo are in an activated state (5). In their role as first responders, neutrophils have several critical functions that contribute to their effectiveness in combating infections and managing inflammation. They generate reactive oxygen species (ROS), which are powerful agents capable of destroying invading pathogens but can also cause tissue damage if uncontrolled. Neutrophils secrete a variety of proteases that help in breaking down cellular debris and pathogens. Additionally, they can release structures known as neutrophil extracellular traps (NETs), which are composed of DNA fibers coated with antimicrobial proteins that trap and kill microbes. Beyond these, neutrophils may function as antigen-presenting cells to activate other immune cells and produce pro-inflammatory cytokines and chemokines that amplify inflammatory responses (35). In vitiligo, the balance of immune cells is disrupted, as evidenced by an increased neutrophil-to-lymphocyte ratio compared to healthy individuals. This altered ratio is significant because it may indicate an overall heightened inflammatory state within the body. Moreover, the increased production of reactive oxygen species by neutrophils in vitiligo patients suggests that these cells are not only more active but also potentially contributing to the oxidative stress that might drive the destruction of melanocytes. This excessive ROS production can further damage cells and exacerbate the depigmentation process characteristic of vitiligo, highlighting the importance of understanding and potentially regulating neutrophil activity in managing the disease (36, 37).

Although our study indicates a significant relationship between circulating immune cells and vitiligo in a large population, there are several limitations. Firstly, our cohort study and MR analysis are based solely on European populations, and caution should be exercised when applying these conclusions to other ethnic groups. Secondly, in our study, we primarily focused on 6 types of circulating immune cells; however, the subtypes of these cells can be further divided, such as neutrophils, which can be categorized into immature and mature cells, with mature cells potentially overlapping with eosinophils. Thirdly, MR analysis is limited to inferring causality and cannot fully explain the causal relationship between the study population and disease severity or duration. Future studies could explore a wider range of immune cells and their interactions to further investigate this relationship. Furthermore, different types of vitiligo are characterized by distinct immune microenvironments. Segmental vitiligo (SV) and non-segmental vitiligo (NSV) are influenced by different pathophysiological pathways. While NSV is associated with increased systemic immune activation, SV does not exhibit this trait (38). However, our vitiligo dataset does not differentiate between these subtypes, which may compromise the reliability of our findings. Lastly, we identified an association between circulating white blood cells and vitiligo; however, the specific mechanisms of circulating white blood cells remain to be further explored.

## 5 Conclusion

In conclusion, our study establishes a causal link between genetically associated circulating immune cells and vitiligo. The presence of elevated circulating white blood cells may serve as a potential risk factor for vitiligo, providing valuable guidance for potential prevention and treatment strategies. To solidify this specific association, further investigation through large-scale randomized controlled trials and detailed scientific animal studies is essential.

## Data availability statement

The original contributions presented in the study are included in the article/**Supplementary Material**. Further inquiries can be directed to the corresponding author.

## Ethics statement

Ethical approval was not required for the studies involving humans because all data are from published publicly available studies, so no additional ethical review is required. The studies were conducted in accordance with the local legislation and institutional requirements. The human samples used in this study were acquired from primarily isolated as part of your previous study for which ethical approval was obtained. Written informed consent to participate in this study was not required from the participants or the participants’ legal guardians/next of kin in accordance with the national legislation and the institutional requirements.

## Author contributions

YX: Writing – review & editing, Writing – original draft, Visualization, Validation, Supervision, Software, Resources, Project administration, Methodology, Investigation, Formal analysis, Data curation, Conceptualization. TY: Writing – review & editing. JW: Writing – review & editing.
