# Supplementary figures and images for: Circulating immune cells and vitiligo: a bidirectional two-sample Mendelian randomization study
Source: Front Immunol. 2024 Jun 3;15:1391186. doi: 10.3389/fimmu.2024.1391186 (PMC11180719; doi:10.3389/fimmu.2024.1391186)

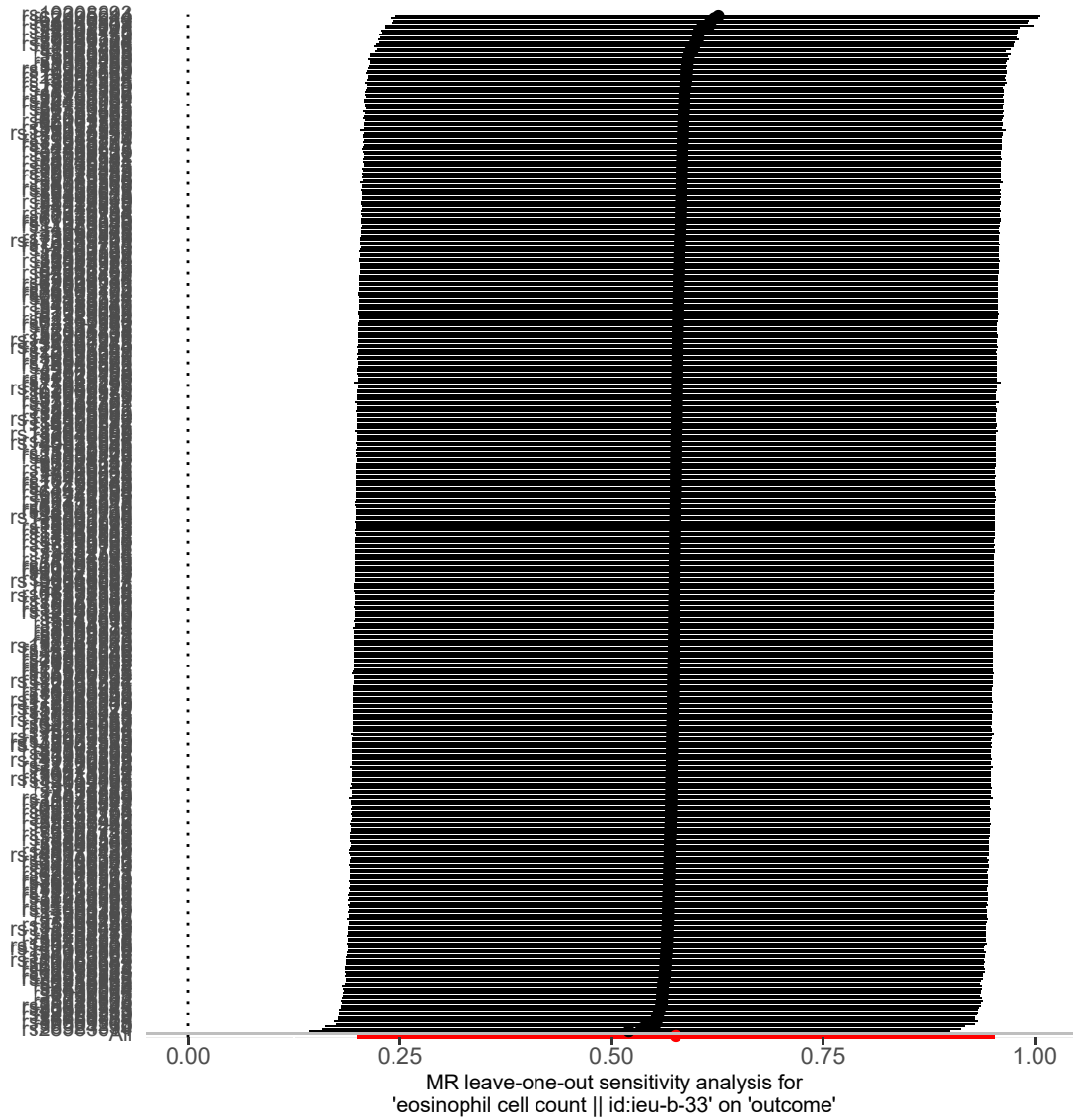

Supplement: Supplementary file 1 [file DataSheet_1.pdf]

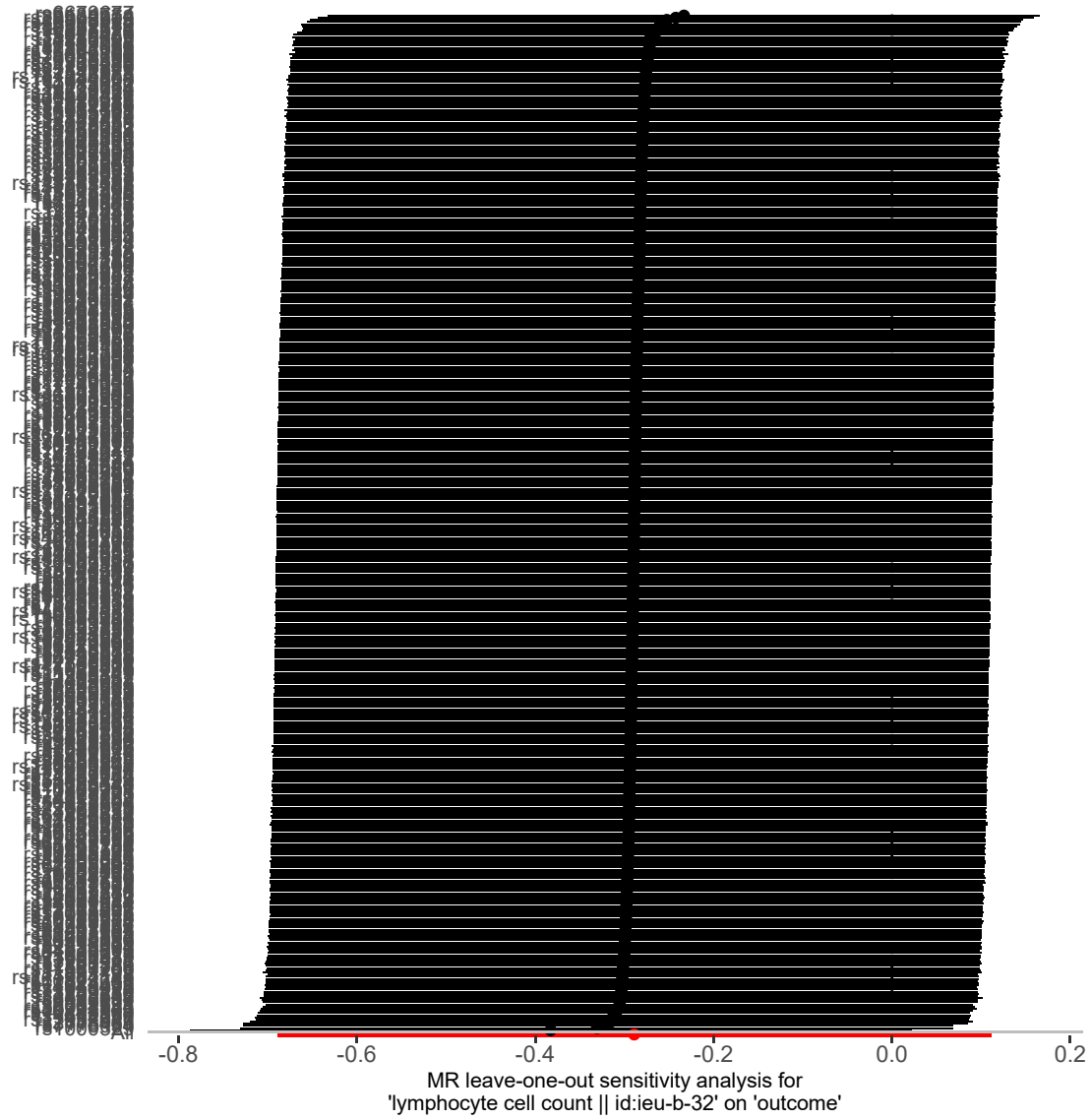

Supplement: Supplementary file 2 [file DataSheet_2.pdf]

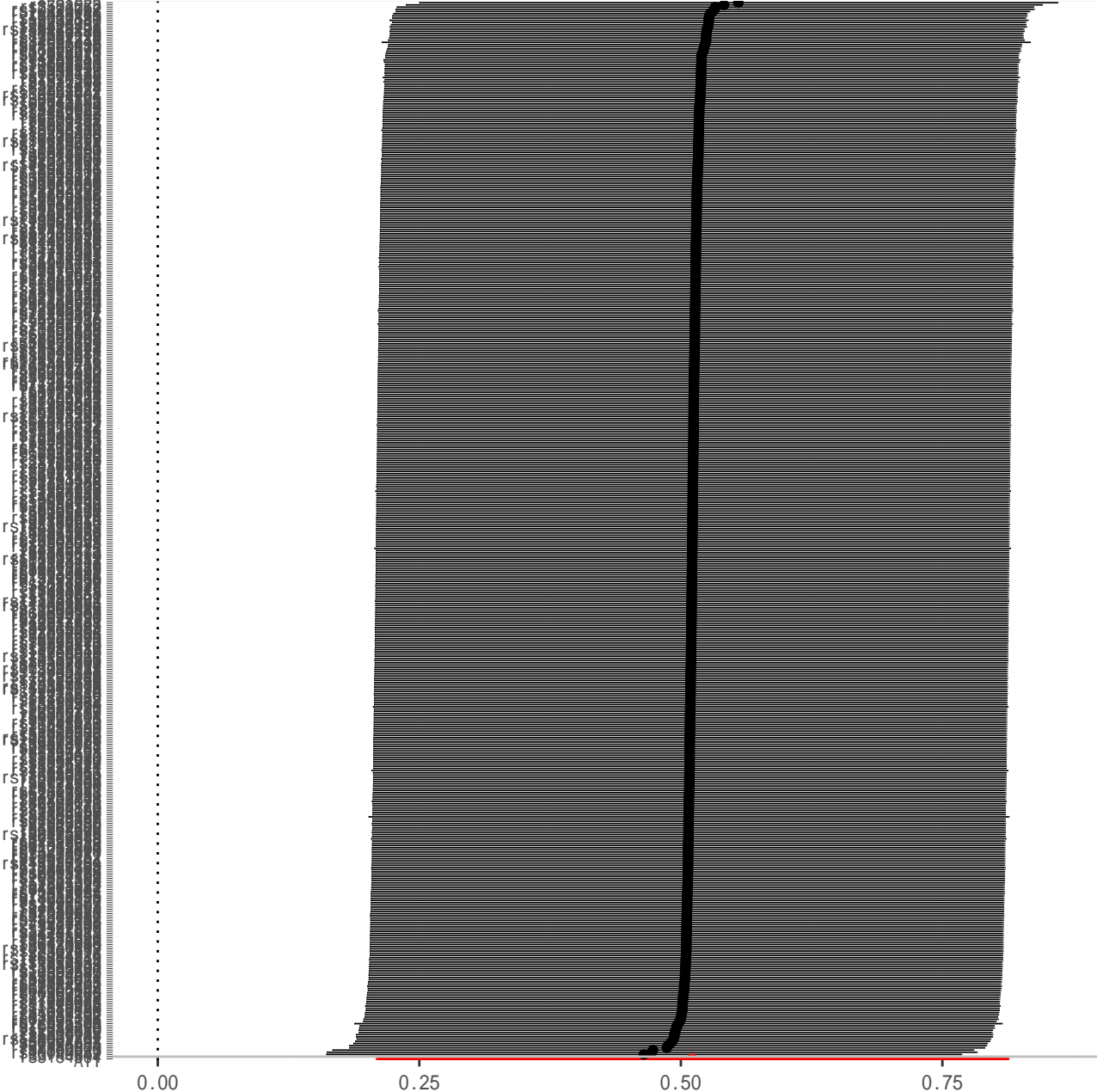

MR leave-one-out sensitivity analysis for  
'monocyte cell count || id:ieu-b-31' on 'outcome'

Supplement: Supplementary file 3 [file DataSheet_3.pdf]

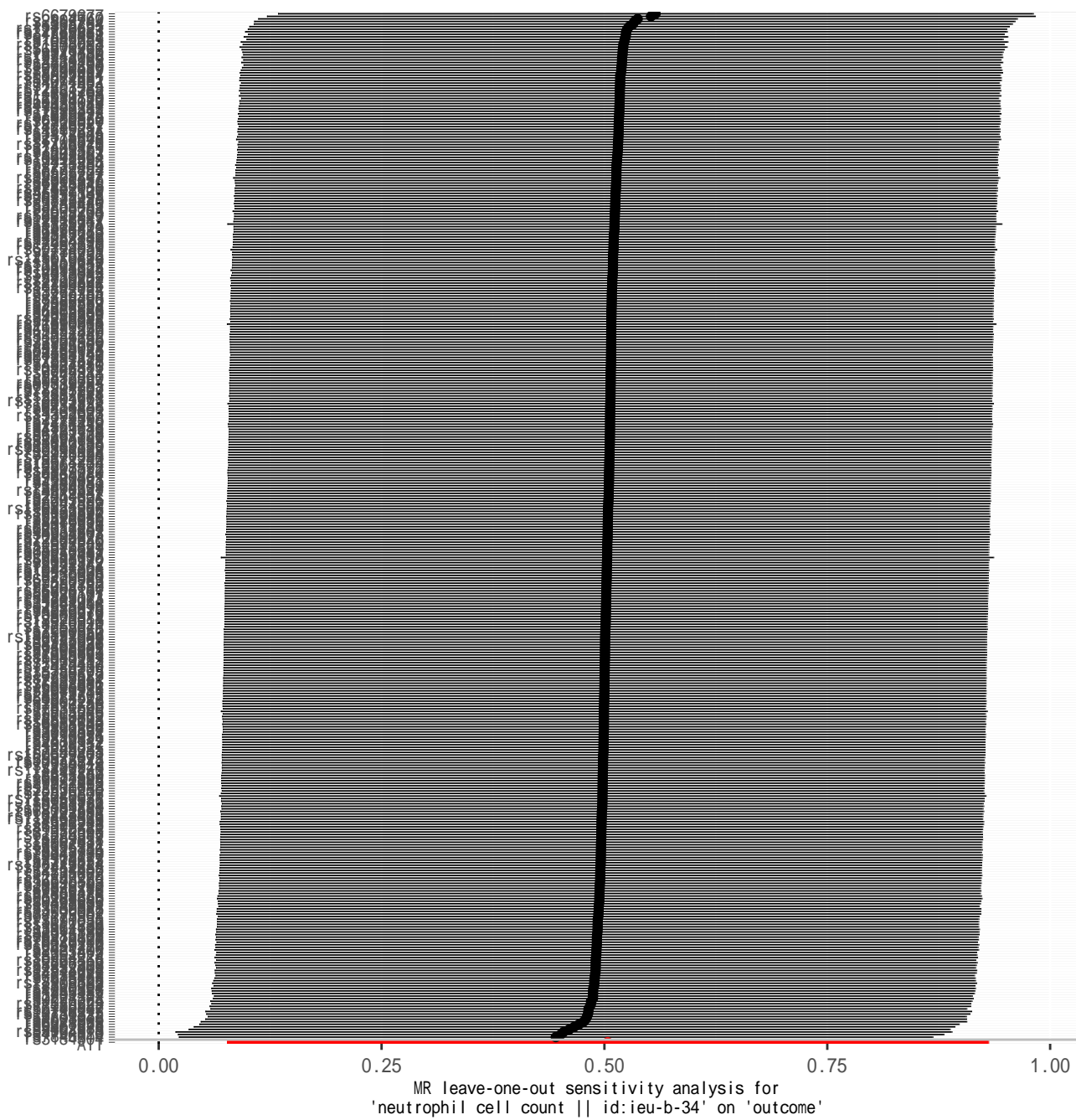

Supplement: Supplementary file 4 [file DataSheet_4.pdf]

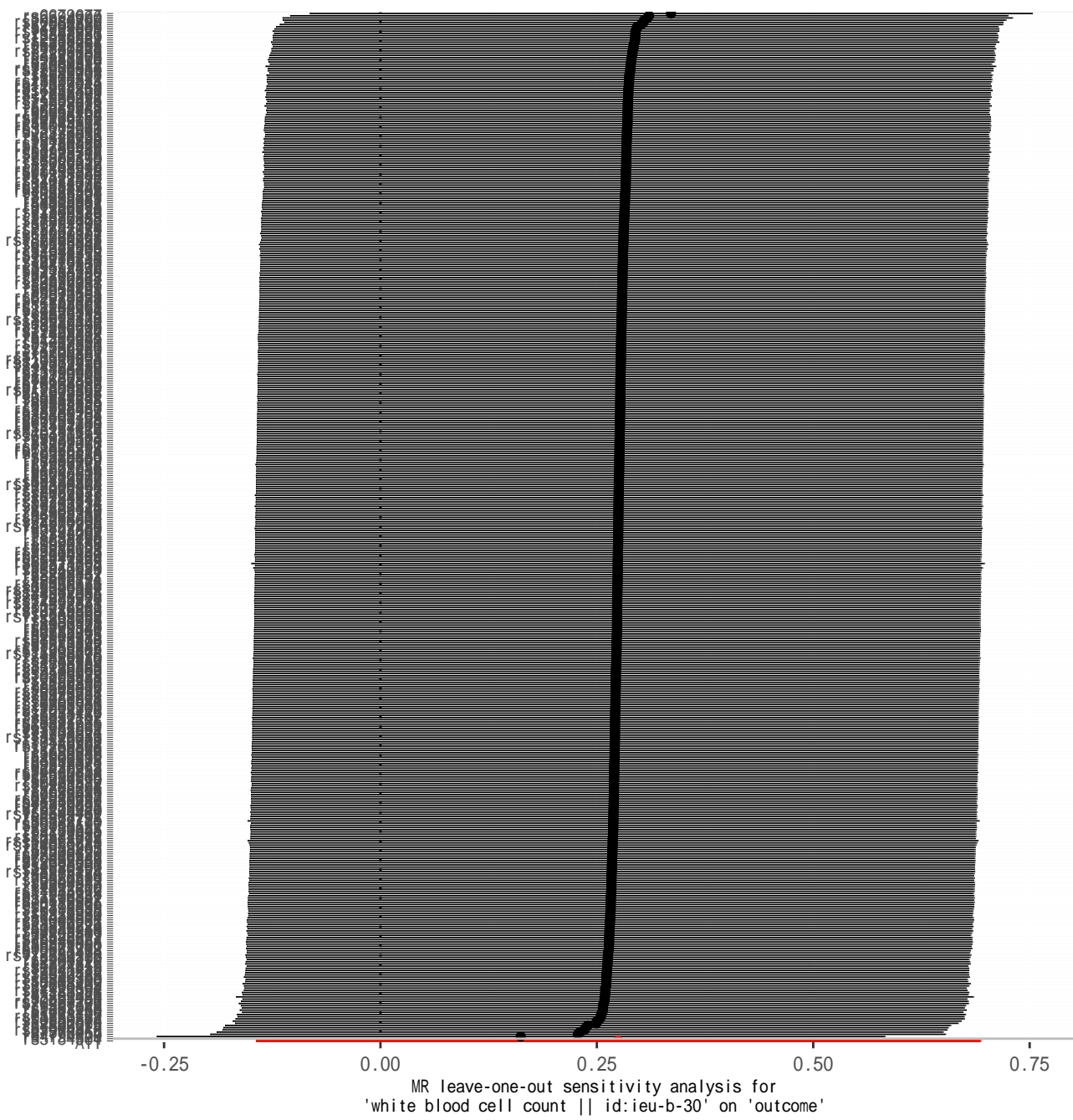

Supplement: Supplementary file 5 [file DataSheet_5.pdf]

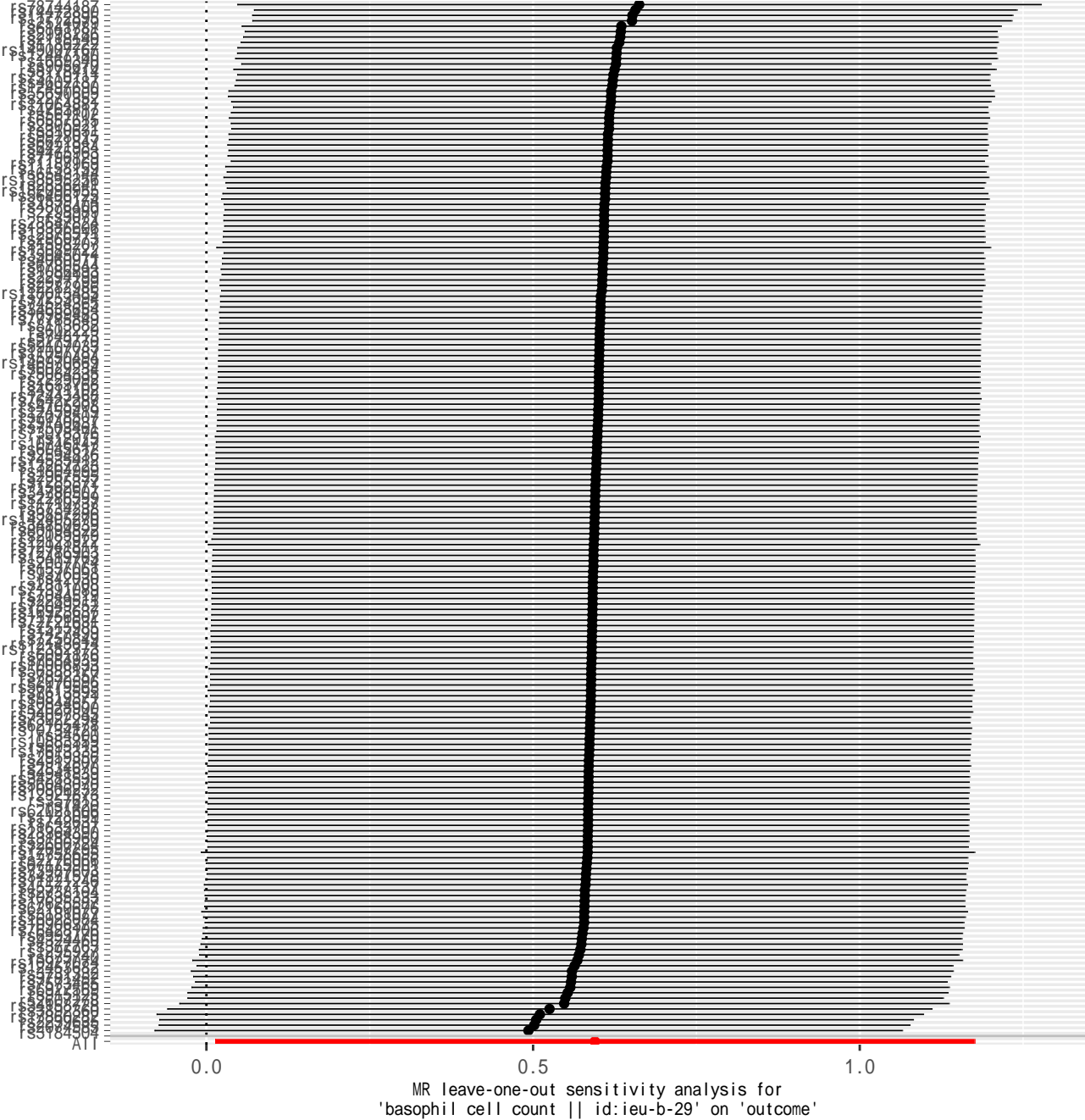

Supplement: Supplementary file 6 [file DataSheet_6.pdf]
