# Supplementary material for: Circulating immune cells and vitiligo: a bidirectional two-sample Mendelian randomization study
Source: Front Immunol. 2024 Jun 3;15:1391186. doi: 10.3389/fimmu.2024.1391186 (PMC11180719; doi:10.3389/fimmu.2024.1391186)
Supplement: Supplementary file 9 [file Presentation_3.pptx]

## Slide 1
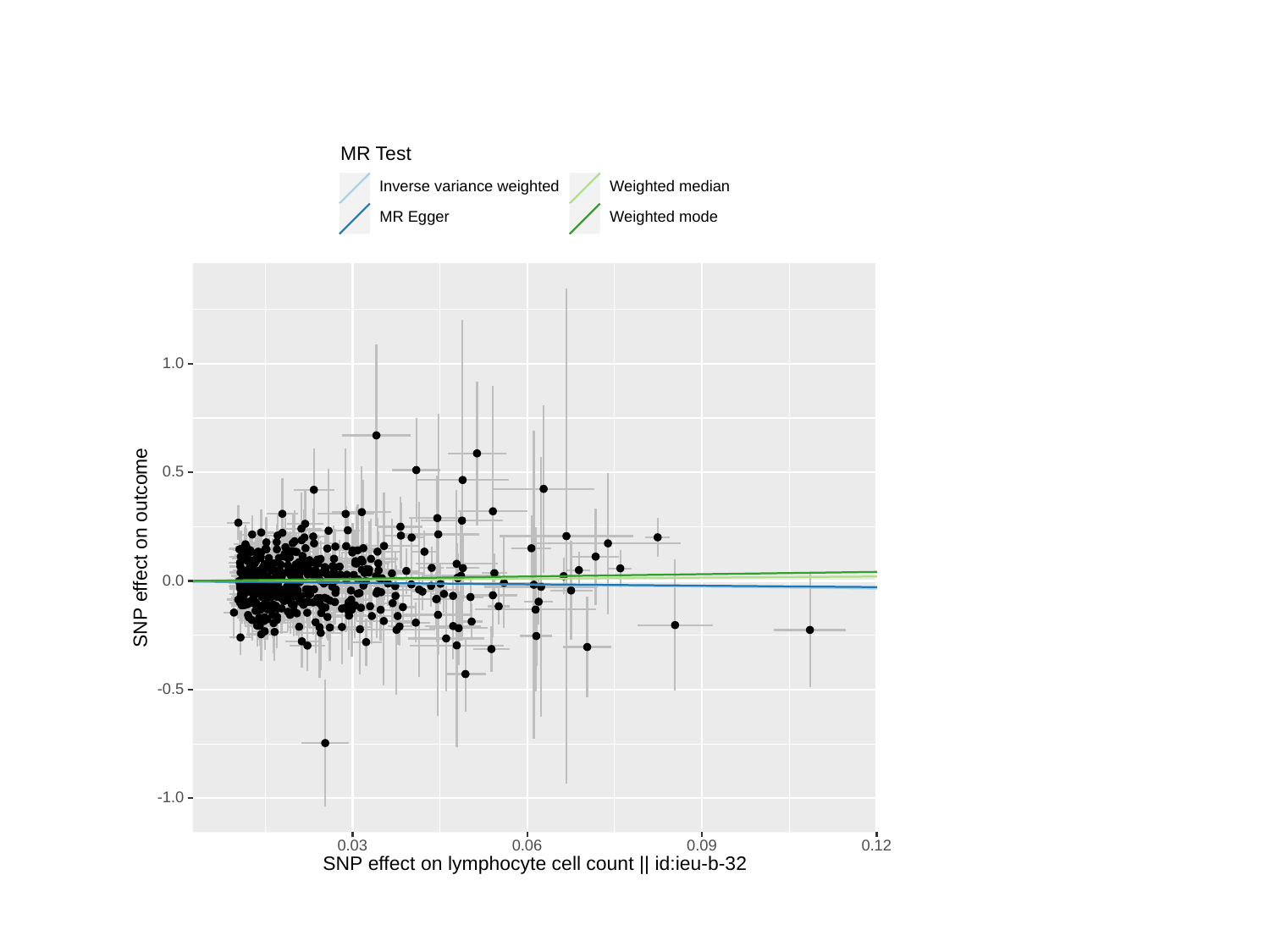

MR Test
Inverse variance weighted
Weighted median
MR Egger
Weighted mode
1.0
0.5
SNP effect on outcome
0.0
-0.5
-1.0
0.03
0.06
0.09
0.12
SNP effect on lymphocyte cell count || id:ieu-b-32
