# Supplementary material for: Circulating immune cells and vitiligo: a bidirectional two-sample Mendelian randomization study
Source: Front Immunol. 2024 Jun 3;15:1391186. doi: 10.3389/fimmu.2024.1391186 (PMC11180719; doi:10.3389/fimmu.2024.1391186)
Supplement: Supplementary file 12 [file Presentation_6.pptx]

## Slide 1
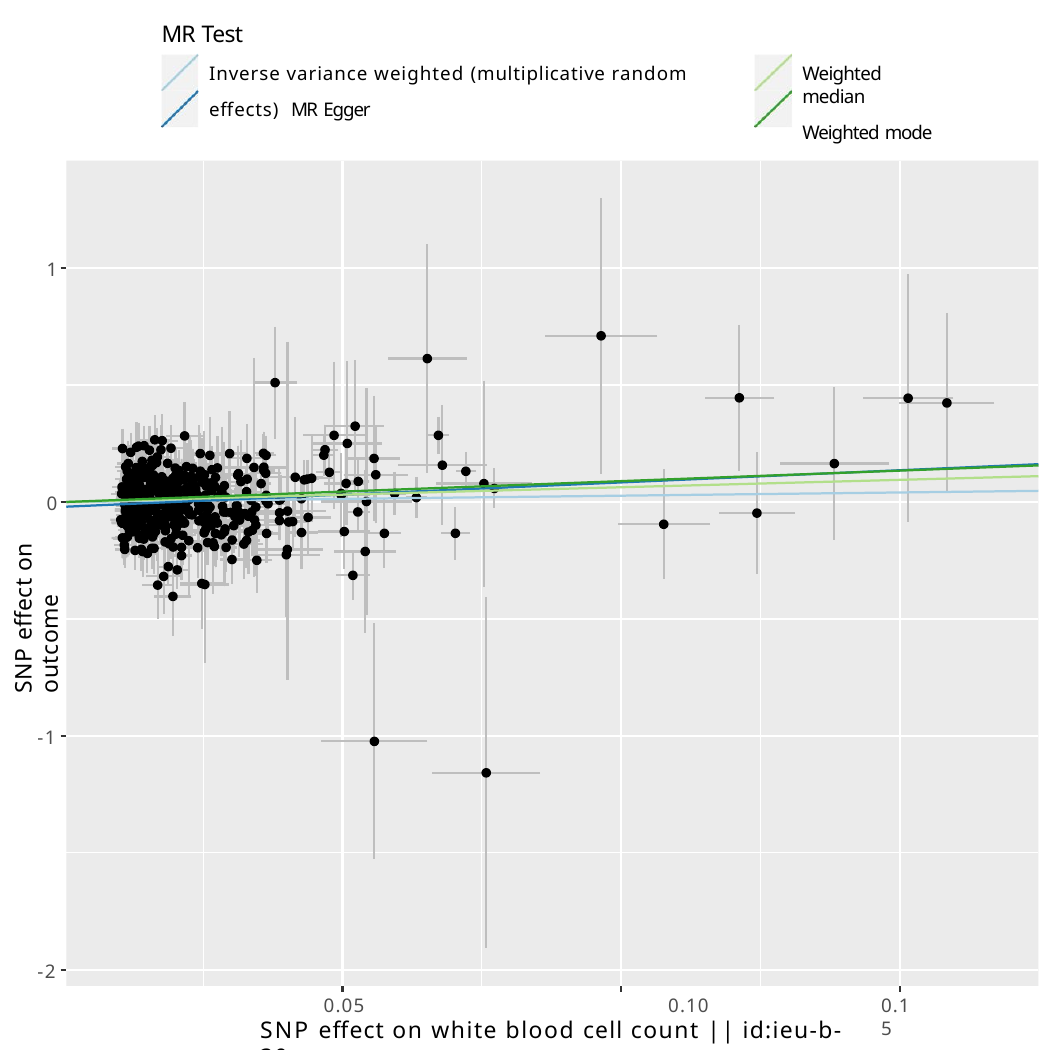

MR Test
Inverse variance weighted (multiplicative random effects) MR Egger
Weighted median
Weighted mode
1
SNP effect on outcome
0
-1
-2
0.05	0.10
SNP effect on white blood cell count || id:ieu-b-30
0.15
